# Comprehensive Characterization of Bioactive Properties in Extracts from Different Chilean Hop Ecotypes (*Humulus lupulus* L.): Antioxidant, Antimicrobial and Antitumor Activities

**DOI:** 10.3390/antiox14101224

**Published:** 2025-10-11

**Authors:** María C. Betancur, Fernando Salazar, Michael Araya, Anita Behn, Jéssica López, Ana Quesille-Villalobos, José M. Villatoro, Jacqueline Poblete, Angara Zambrano

**Affiliations:** 1 Escuela de Alimentos, Facultad de Ciencias Agronómicas y de los Alimentos, Pontificia Universidad Católica de Valparaíso, Waddington 716, Playa Ancha, Valparaíso 2362807, Chile; carolinabetancurda@gmail.com; 2 Laboratorio de Fermentaciones Industriales, Escuela de Alimentos, Facultad de Ciencias Agronómicas y de los Alimentos, Pontificia Universidad Católica de Valparaíso, Waddington 716, Valparaíso 2362807, Chile; fernando.salazar@pucv.cl; 3 Centro de Investigación y Desarrollo Tecnológico en Algas (CIDTA), Facultad de Ciencias del Mar, Universidad Católica del Norte, Larrondo 1281, Coquimbo 1780000, Chile; mmaraya@ucn.cl; 4 Facultad de Ciencias Agrarias y Alimentarias, Instituto de Producción y Sanidad Vegetal, Universidad Austral de Chile, Valdivia 5090000, Chile; anita.behn@uach.cl; 5 Facultad de Medicina Veterinaria y Agronomía, Universidad de Las Américas, Manuel Montt 948, Santiago 7500975, Chile; aquesille@gmail.com; 6 Institute of Pharmacy, Faculty of Sciences, Universidad Austral de Chile, Casilla 567, Valdivia 5090000, Chile; jmvillatoro@uach.cl; 7 Food Engineering Department, Universidad de La Serena, Av. Raúl Bitrán 1305, La Serena 1700000, Chile; j.pobletegalleguillos@gmail.com; 8 Instituto de Bioquímica y Microbiología, Facultad de Ciencias, Universidad Austral de Chile, Valdivia 5090000, Chile; angarahzambrano@gmail.com; 9 Center for Interdisciplinary Studies on the Nervous System (CISNe), Universidad Austral de Chile, Valdivia 5090000, Chile

**Keywords:** Chilean hop ecotypes, *Humulus lupulus*, antioxidant activity, antimicrobial resistance, xanthohumol, antitumor properties

## Abstract

Chilean hop (*Humulus lupulus* L.) ecotypes are an under-explored resource with high bioactive potential, offering promising applications in food preservation and health promotion. This study aimed to characterize the chemical composition and evaluate the antioxidant, antimicrobial, and cytotoxic properties of methanolic extracts from three native ecotypes—*Ranco*, *La Unión*, and *Valdivia*—to identify their potential as sources of multifunctional bioactive compounds. Each ecotype exhibited a distinct composition of bioactive compounds; Valdivia stood out for its pronounced levels of α- and β-acids and xanthohumol. Antioxidant capacity, assessed by DPPH, FRAP, and ABTS, was strong across extracts, with Valdivia showing the highest values in all the tests carried out. The extracts inhibited multidrug-resistant clinical isolates, notably *Enterococcus faecalis* and *Pseudomonas aeruginosa*, and showed dose-dependent cytotoxic effects in H1299 and MCF-7 cell lines, with the La Unión extract particularly active against H1299. Overall, these findings position Chilean hop ecotypes as promising sources of natural antioxidants and antimicrobial agents for functional food and nutraceutical applications.

## 1. Introduction

Hop (*Humulus lupulus*) is a plant traditionally used in the brewing industry for its characteristic sensory properties and preservation effects [1]. Beyond these applications, hop contains bioactive compounds such as phenolic compounds, resins, and essential oils, which provide antioxidant, anti-inflammatory, and antimicrobial benefits, generating increasing interest in the food and pharmaceutical industries [2].

**Figure 1:**
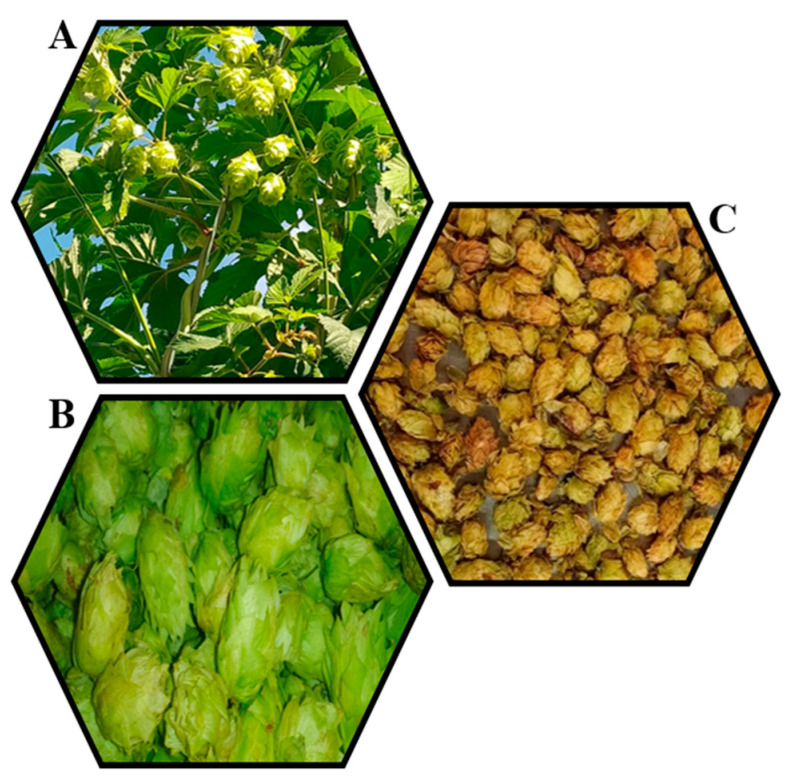
Hop (*Humulus lupulus*) ecotype *Ranco* plant (**A**), fresh cones (**B**), and dried cones (**C**).

Its cultivation has expanded worldwide, and local ecotypes—such as those from Chile—are attracting attention for their biofunctional potential [3,4,5]. In particular, the *Ranco* ecotype (Figure 1), grown in the Los Ríos Region, stands out for its high content of phenolic compounds, which suggests a significant potential for its application in the food and cosmetic industries [6].

Hops are a rich source of secondary metabolites with multiple biological activities. Prenylated flavonoids such as xanthohumol have demonstrated anticancer potential by inducing apoptosis, inhibiting angiogenesis, and suppressing tumor proliferation in various cancer cell lines [7,8]. These findings highlight hops not only as natural preservatives but also as promising sources of therapeutic agents for chronic diseases. In addition, fatty acids present in hop extracts—though less studied—are gaining attention for their role in modulating inflammatory pathways, supporting immune function, and enhancing the bioactivity of other phytochemicals [9].

The antimicrobial potential of hops has been widely documented, particularly against pathogenic and antibiotic-resistant bacteria. Phenolic compounds, including flavonoids and bitter acids, can disrupt bacterial membranes and interfere with essential cellular processes such as protein and DNA synthesis, thereby contributing to their antimicrobial effects [10,11]. However, most research has focused on European and North American cultivars, such as *Cascade* and *Saaz*, which are known for their established profiles of α-acids (typically 3–8%) and β-acids (4–6%) [12,13] and xanthohumol (0.25–0.31% dry weight) [14]. In contrast, Chilean ecotypes, including Valdivia, may present distinct bioactive profiles, highlighting their potential as alternative sources of bioactive compounds beyond traditional cultivars.

This research gap is particularly relevant given the urgent global concern about antimicrobial resistance, which is projected to cause up to 10 million deaths annually by 2050 if effective alternatives are not developed [15,16]. Despite advances in the study of hop bioactivity, limited information is available regarding the phenolic profile, antioxidant capacity, and antimicrobial potential of Chilean ecotypes against multidrug-resistant microorganisms. Addressing this gap is essential to establish their value as innovative natural sources for the health and functional food industries.

Therefore, the objective of this study was to comprehensively evaluate the bioactive properties of methanolic extracts from three Chilean hop ecotypes. Specifically, we analyzed their fatty acid composition, quantified bitter acids and xanthohumol using high-performance liquid chromatography (HPLC), and assessed their antioxidant, antimicrobial, and antitumor activities to provide new insights into their applicability in food and health-related sectors.

## 2. Materials and Methods

### 2.1. Hop Cones Preparation

The cones of the three ecotypes of Chilean hops, *Ranco*, *La Unión*, and *Valdivia* were purchased from Kalfvlup Hops, located in Calfuco, a coastal area of the commune of Valdivia, the Los Ríos Region, Chile, (−39.4918° S, −72.8425° O). Fresh hop cones of the three ecotypes were dried to stabilize and preserve their properties for further analysis. A pilot-scale tray dryer (UOP 8 tray dryer, Armfield, UK) was used at 60 °C at a 1.0 m/s air velocity, and the dehydration process was carried out until the cones were dried to about 95% dry matter (d.m.). Dried hop cones were finely ground in a mill (IKA A-11, Wilmington, NC, USA) and thereafter sieved through a 500 μm sieve to obtain homogeneous particles. The sieved powders were stored under vacuum and in darkness at −20 °C before analysis.

### 2.2. Extraction Procedure

A conventional solvent extraction was performed from a previous work [6], with some modifications. Two hundred and fifty milligrams of pulverized dried cones were mixed with methanol: water (90:10, *v*/*v*) adjusted to pH 2.5 by phosphoric acid in the ratio 1:100 (*w*/*v*). The extraction was carried out at room temperature for 60 min in an orbital shaker (VS-201D; Vision Scientific Co., Ltd., Daejeon, Republic of Korea) at 250 rpm. The extracts were centrifuged (Model Z 326 K; Hermle Labortechnik, Wehingen, Germany) at 2470× *g* for 10 min, and the supernatants obtained were filtered through a syringe filter (Whatman Uniflo, GE Healthcare, Chicago, IL, USA, 25 mm, 0.45 μm) into a 25 mL volumetric flask and brought to volume with the extraction solvent. Extractions were performed in triplicate.

### 2.3. Proximate Analysis

The different hop ecotypes were subjected to proximate analysis, considering the content of moisture (NCh 841:2018), fat (NCh3547:2018), protein (AOAC no. 928.08), ash (AOAC no. 923.03), and total carbohydrates were estimated by difference. All analyses were performed in triplicates.

### 2.4. Determination of the Amino Acid Profile

The quantification of amino acids was determined using an HPLC-DAD MD-2015 Plus pre-column derivatization method adapted from Araya et al. [17]. First, 6 N HCl (10 mL) was added to semi-capped hydrolysis tubes containing the sample (200 mg), which were incubated for 24 h at 120 °C in an oven. Then, the solution was transferred to a volumetric flask with a capacity of 50.0 mL and adjusted with distilled water. A 100 μL aliquot of the solution was extracted and adjusted to pH 10.0 within a 1.0 mL borate buffer and concentrated to dryness in a rotary evaporator. The concentrated extracts were reconstituted to a volume of 200 μL with borate buffer (pH 10.0) and filtered using a 0.22 μm Nylon syringe filter for their derivatization. The amino acids were derivatized with o-phthalaldehyde (OPA) before injection in the autosampler Jasco AS-2055. In step 1, 20 μL of standard mix or sample and 30 μL of borate buffer, pH 10.2, were mixed. In step 2, the previous mix was treated with air and then added to 20 μL of OPA. In step 3, the mixture was deposited in a mixing vial and homogenized with air. Finally, an aliquot was taken from the mixing vial and injected into the equipment. The chromatographic conditions were as follows: flow rate 2.0 mL min−1, the ramp begins at 0–1.9 min, 100% (A); 18.1–18.6 min, 42% (A) 58% (B); 22.3 min, 30% (A) 70% (B); 22.40–26.00 min, 100% (C); 26.10–28.00 min, 100% (A). Separation was performed on a reversed phase column ZORBAX Eclipse AAA (150 × 4.6 mm, 3.5 μm) from Agilent (Santa Clara, CA, USA) thermostated at 40 °C. The injection volume was 10 μL, and the chromatographic run time was set as 28 min. The detection was performed by recording the spectra between 240 nm and 400 nm. The measurement was made at 338 nm using external calibration curves prepared for each amino acid, with regression coefficients (R^2^) ≥ 0.99. The results were expressed as percentages of g/100 g dry matter (d.m.)).

### 2.5. Determination of the Fatty Acid (FA) Profile

A lipidic extract prepared by weighing a 5 g sample of hop was mixed with 40 mL methanol and 20 mL chloroform with Ultraturrax (Polytron PT 2500 E, Malters, Switzerland) for 2 min, then adding 20 mL chloroform and stirring for 30 s, and continued with 20 mL water for 30 s. The extracts were centrifuged 10 min at 4193× *g*, the lipid fraction was washed with hexano, and the lipid extract was recovered. 25 mg of hop extract was weighed and mixed with 500 μL of hexane. Saponification was performed with a 50 μL aliquot of the hop extract and mixed with 200 μL KOH/methanol (2 N) and vortexed for 1 min. Then N-hexane was used to extract the fatty methyl acid by adding 500 µL and stirring for 3 min, and allowed to stand for 1 h. Fatty acid methyl esters (FAME) were injected into a gas chromatograph (Shimadzu, model CG-2010, Shimadzu Corporation, Kyoto, Japan) equipped with an autosampler and a flame ionization detector (FID), operating at 250 °C according to Quispe-Fuentes et al. [18]. Separation was carried out on a BPX-90 capillary column (100 m × 0.25 mm ID, 0.25 μm film thickness; SGE Analytical Science, Melbourne, Australia). The chromatographic conditions were as follows: the oven temperature was initially held at 100 °C for 13 min, then increased to 180 °C at a rate of 10 °C/min and maintained for 6 min. Subsequently, the temperature was raised to 200 °C at a rate of 1 °C/min and held for 20 min, followed by a final increase to 230 °C at a rate of 4 °C/min and held for 7 min. A 1 μL aliquot of the FAME sample was injected in split mode. Fatty acids were identified by comparing their retention times with those of a standard commercial FAME mixture containing 37 components. The results were expressed as percentages of saturated fatty acids (SFA), monounsaturated fatty acids (MUFA), and polyunsaturated fatty acids (PUFA).

### 2.6. Determination of Bitter Acids and Xanthohumol

The α-acids and β-acids, as well as xanthohumol, were separated and quantified using an HPLC-DAD system (Shimadzu, Kyoto, Japan). Before analysis, extracts from the different hop ecotypes were filtered through Whatman^®^ Uniflo^®^ syringe filters (25 mm, 0.45 μm). The HPLC method was adapted from the ASBC (American Society of Brewing Chemists) Hops-14 method [19]. Detection was carried out at 327 nm for α- and β-acids, and at 368 nm for xanthohumol. Peak integration was performed using LabSolutions software version 5.91 (Shimadzu, Japan). All analyses were conducted in triplicate. The results were expressed as weight percentage on a dry matter basis.

### 2.7. Antioxidant Capacity

The antioxidant capacity was measured by DPPH (2,2-Diphenyl-l-picrylhydrazyl radical), FRAP (ferric reducing antioxidant capacity), and ABTS (2,2′-azino-bis-3-ethylbenzthiazoline-6-sulphonic acid) radical cation assays.

#### 2.7.1. DPPH Assay

The DPPH radical scavenging activity of hop extracts was measured using a previously described method [20], adapted for microplate assays. 100 µL of 60 µM methanolic solution of DPPH was added to 100 µL of hop extracts and incubated for 30 min in the dark. The absorbance was measured at 517 nm in a spectrophotometer (Multiskan Go, Thermo Scientific, Waltham, MA, USA) and compared to a Trolox equivalent (TE) calibration curve (Y = 0.5714X + 11.397, R^2^ = 0.9576). The results were expressed as mM Trolox equivalents (TE) per g (mM TE/g of dry matter).

#### 2.7.2. FRAP Assay

The FRAP assay was performed following a previously published method, with some modifications [21]. The ferric tripyridyltriazine (Fe(III)-TPTZ) complex was prepared by mixing 25 mL of sodium acetate buffer (pH 3.6), 2.5 mL of TPTZ solution, and 2.5 mL of FeCl_3_ (20 mmol/L). 15 µL of hop extracts was mixed with 285 µL of Fe(III)–TPTZ complex, incubated for 30 min in the dark at 37 °C, and the absorbance measured at 593 nm in a spectrophotometer (Multiskan Go, Thermo Scientific, Waltham, MA, USA) and compared to a Trolox equivalents (TE) calibration curve (Y = 0.0016X − 0.0493, R^2^ = 0.9831). The results were expressed as mM Trolox equivalents (TE) per g (mM TE/g of dry matter).

#### 2.7.3. ABTS Assay

The ABTS assay was carried out using the method described by Maliar et al. [21] with slight modifications. The ABTS radical cations (ABTS+) were prepared using an ABTS stock solution (7 Mm) and a potassium persulphate stock solution (4.9 mM) adjusted to 10 mL with distilled water and mixed in a 1:1 ratio. The mixture was left to stir for 16 h in the dark at room temperature, and then the absorbance was adjusted to 0.70 with methanol at 734 nm. One hundred microliters of ABTS+ working solution was mixed with one hundred microliters of the hop extracts and incubated for 7 min in the dark. The absorbance was measured at 734 nm in a spectrophotometer (Multiskan Go, Thermo Scientific, Waltham, MA, USA) and compared to a Trolox equivalents (TE) calibration curve (Y = 2.1793X + 1.1978, R^2^ = 0.9985). The results were expressed as mM Trolox equivalents (TE) per g (mM TE/g of dry matter).

### 2.8. Antimicrobial Activity

#### 2.8.1. Strains and Characterization

Three multidrug-resistant Gram-negative isolates—*Klebsiella pneumoniae*, *Escherichia coli*, and *Pseudomonas aeruginosa*—resistant to third- and fourth-generation cephalosporins and/or carbapenems, and one vancomycin-resistant *Enterococcus faecalis* isolate were selected from the collection of the Genomics & Resistant Microbes Laboratory (GeRM) at ICIM–Universidad del Desarrollo, Chile. All isolates were collected from Chilean hospitals. Species identification was performed using MALDI-TOF (Bruker), and antimicrobial resistance was confirmed by disk diffusion assay following the Clinical and Laboratory Standards Institute guidelines [22].

#### 2.8.2. Minimal Inhibitory Concentration (MIC)

Antibacterial activity was assessed using multidrug-resistant bacteria isolated from patients with bacteremia, along with American Type Culture Collection (ATCC) strains as a control. The antimicrobial effects of all hop extracts were measured using the MIC through the broth microdilution method, following the Clinical and Laboratory Standards Institute [22] recommendations with some modifications for the hop extracts. Briefly, the extracts were diluted 1:1 in Mueller–Hinton broth and then mixed with an equal volume of bacterial suspension, achieving a final concentration of 10^5^ CFU/mL. Both sterility and growth controls were included in the experiment. The plates were incubated at 37 °C for 18 h on a shaker set to 150 rpm. The MIC is defined as the lowest concentration that inhibits visible bacterial growth.

### 2.9. Anti-Tumoral Activity

#### 2.9.1. Cell Culture

The cell line H1299 (ATCC: CRL-5803™) was maintained in cell culture bottles at 37 °C with 5% CO_2_ in RPMI-1640 medium supplemented with 10% fetal bovine serum (FBS, Sigma-Aldrich, St. Louis, MO, USA) and 1% penicillin/streptomycin. The cell line MCF-7 (ATCC: HTB-22) was maintained in similar conditions, in Dulbecco’s Modified Eagle’s Medium (DMEM; Sigma-Aldrich, St. Louis, MO, USA) medium supplemented with 10% FBS, 1% penicillin/streptomycin and 2 mM L-glutamine.

#### 2.9.2. Cell Viability

H1299 and MCF-7 cells were seeded in 96-well plates (5000 cell/well). The next day, cells were treated with different concentrations, from 0 to 10 mg/mL of the different hop extracts. After 48 h of treatment, the cell viability was evaluated by methyl thiazol tetrazolium (MTT) assays. Four hours before the end of treatment a solution of 0.5 mg/mL of MTT was added to the cells and finally, the absorbance was measured at 570 nm with a reference wavelength of 690 nm. Each extract was evaluated in triplicate, and each experiment was repeated at least 3 times. The data were expressed as the mean ± standard error of the mean (SEM).

### 2.10. Statistical Analysis

Statistical analysis was performed using Statgraphics Centurion XVIII program (Statistical Graphics Corp., Herndon, VA, USA) using analysis of variance (one-way ANOVA). The significant differences (*p* < 0.05) between the means were performed using the least significant difference (LSD) test at a significance level of α = 0.05 and a confidence interval of 95% (*p* < 0.05). A multiple-range test (MRT) was also performed to demonstrate the existence of homogeneous groups within each of the parameters.

## 3. Results and Discussion

### 3.1. Proximate Characterization

The results of the proximate composition of the Chilean hop ecotypes *Ranco*, *La Unión*, and *Valdivia* are presented in Table 1. These were evaluated to identify significant differences in terms of moisture, ash, fat, protein, and total carbohydrates. It was found that the *Ranco* ecotype presented the highest moisture content (5.45 ± 0.01% d.m.), significantly higher than that of *La Unión* (4.52 ± 0.04% d.m.) and *Valdivia* (3.63 ± 0.02% d.m.), reflecting possible differences in water retention capacity. In terms of ash, *Valdivia* showed the highest percentage (8.05 ± 0.09% d.m.), while *Ranco* presented the lowest value (7.39 ± 0.76% d.m.). *La Unión* stood out for its fat content (2.37 ± 0.10% d.m.), significantly exceeding *Valdivia* (1.94 ± 0.04% d.m.) and *Ranco* (1.90 ± 0.02% d.m.). Regarding protein, *La Unión* also presented the highest percentage (14.02 ± 0.26% d.m.), followed by *Ranco* and *Valdivia*; however, the results are comparable to those reported by Almaguer et al. [23] for whole and dry hop cones, which indicate a general average is 15%. Finally, *Valdivia* showed the highest total carbohydrate content (78.90 ± 0.18% d.m.), significantly higher than *Ranco* and *La Unión* which, compared to a related study [24] in which hop pellets of *Perle Hallertau* and *Nugget* varieties were evaluated, showed carbohydrates contents of about 30%, suggesting that Chilean hops contain high levels of total carbohydrates.

Differences in proximate composition may reflect the influence of agricultural practices within the Los Ríos Region. In the case of the *Valdivia* ecotype, the high carbohydrate levels observed may be related to the specific climatic conditions of this locality, which favor sugar accumulation [25].

It was expected not to observe significant variations in all the parameters evaluated due to the differences in the ecotypes, as Behn et al. [26] suggest that the three Chilean ecotypes were genetically identical considering the single-nucleotide polymorphism (SNP) in the high polymorphic A1 region [27]; however, the high levels of ash and total carbohydrates in *Valdivia*, the high levels of protein and fat in *La Unión* demonstrate the potential that Chilean hop ecotypes can have as sources of nutrients.

### 3.2. Amino Acid Profile

In total, 15 amino acids were identified from the three ecotypes of Chilean hops determined by HPLC methodology. Table 2 shows the content of amino acids present in the samples, as well as the total sum of each amino acid. In general, aspartic acid (ASP) and cystine (CY2) were the most abundant, with the highest levels observed in *Ranco* (ASP: 1.80 ± 0.26 g/100 g d.m.; CY2: 1.50 ± 0.23 g/100 g d.m.). Overall, the concentrations measured in the analyzed samples are higher than those reported by Gahr et al. [28] for five commercial cultivars (*Huelle Melon*, *Saphir*, Hers*brucker*, *Herkules* and *Amarillo*), although 19 amino acids were analyzed, the sum of these corresponds to 2.3 g/100 g of dry hop sample, while in this work the *Ranco* ecotype presented more than 7 g/100 g, which could be related to a greater capacity of this ecotype to generate bioactive compounds effective against resistant bacteria.

In a recently published study [29], where changes in the concentration of amino acids during fermentation of hop beer were studied, the presence of amino acids in dry hops was evaluated, with asparagine and glutamine found in the highest concentrations. It has been shown that amino acids are precursors of metabolic pathways involved in the biosynthesis of phenolic compounds and antioxidants, which in turn inhibit the growth of resistant bacteria by altering the cell membrane and its oxidative processes [30]. Beyond this metabolic role, amino acids have been used to increase the efficacy of existing drugs, improving their efficiency by a synergistic effect, inhibiting the growth of some microorganisms [31]. For example, the incorporation of amino acids into bactericidal formulations has been associated with reductions of approximately 2 to 3 log CFU have been exhibited [32]. In the case of glutamine [33], a study was able to demonstrate that this amino acid favored the death of *E. coli* caused by β-lactams, aminoglycosides, quinolones and tetracyclines. Likewise, glutamine potentiated the activity of ampicillin against clinically relevant pathogens such as *Pseudomonas aeruginosa*, *Acinetobacter baumannii*, *Klebsiella pneumoniae*, *Edwardsiella tarda*, *Vibrio alginolyticus* and *Vibrio parahaemolyticus*. Furthermore, glycine has demonstrated bactericidal activity on multidrug-resistant bacteria and restored the susceptibility of some multidrug-resistant nosocomial pathogens to antibiotics [34].

Among ecotypes, significant differences were observed in lysine (LYS), where *Ranco* showed a higher content (0.77 ± 0.05 g/100 g d.m.) compared to *La Unión* (0.55 ± 0.02 g/100 g d.m.) and *Valdivia* (0.48 ± 0.10 g/100 g d.m.). Lysine has been associated with the synthesis of alkaloids and other secondary metabolites that may exert an antimicrobial activity [35]. Microorganisms have developed tactics to resist antimicrobials by decreasing their efficacy or neutralizing them [36]. This occurs due to protein channels in the microbial cell envelope, called porins, which promote the transport of substances. In some microorganisms, due to the composition of the membrane, the passage of drugs is hindered, reducing the concentration inside the cell, avoiding lethal effects [37].

Although there are no statistically significant differences for most amino acids between hop ecotypes, the results emphasize the potential of each ecotype as a source of specific nutritional compounds. The variability in the amino acid composition of Chilean hop ecotypes and their high concentrations, especially in the *Ranco* ecotype, not only highlights their potential as a nutritional source but also suggests that these compounds could be essential in the synthesis of secondary metabolites with antimicrobial properties.

### 3.3. Fatty Acid (FA) Profile

The lipid profile analysis of three Chilean hop ecotypes (*Ranco*, *La Unión*, and *Valdivia*) enabled the characterization of major saturated (SFA), monounsaturated (MUFA), and polyunsaturated fatty acids (PUFA), as presented in Table 3. This characterization provides evidence of the functional and nutraceutical potential of these compounds in the evaluated plant extracts.

Among all ecotypes, three predominant saturated fatty acids were identified: undecanoic, palmitic, and stearic acids. Palmitic acid was the most abundant, ranging from 30.67 ± 1.26% d.m. in *Valdivia* to 33.85 ± 1.48% d.m. in *La Unión*, with no statistically significant differences among ecotypes (*p* > 0.05). Stearic acid content was highest in *Ranco* (19.88 ± 0.53% d.m.) compared to *Valdivia* (16.12 ± 1.70% d.m.), while undecanoic acid levels were consistent across ecotypes (~5%). Overall, total SFA content was highest in *La Unión* (56.35 ± 2.23% d.m.), followed by *Ranco* (53.22 ± 0.35% d.m.) and *Valdivia* (51.86 ± 2.91% d.m.).

Regarding unsaturated fatty acids, linoleic acid (PUFA) and eicosenoic acid (MUFA) were the most representative. *Valdivia* exhibited the highest eicosenoic acid content (35.83 ± 2.45% d.m.), with values significantly exceeding those of *Ranco* (31.41 ± 0.83% d.m.) and *La Unión* (31.86 ± 1.74% d.m.), while linoleic acid levels remained similar across all ecotypes (~15–17%). The total MUFA and PUFA content was highest in *Valdivia*, followed by *La Unión* and *Ranco*, suggesting a higher proportion of functional fatty acids in the *Valdivia* ecotype.

Although no statistically significant differences (*p* > 0.05) were detected among ecotypes in terms of fatty acid content, the compositional trends observed suggest lipid profiles with potential implications for nutraceutical development, these findings provide a novel perspective on the nutritional quality of hop-derived lipids, particularly in landrace-like varieties such as the Chilean ecotypes evaluated.

These findings are partially consistent with those reported by Farag et al. [38] who identified palmitic, linoleic and linolenic acids as predominant fatty acids in hop extracts obtained using ethanol. Additionally, Rettberg et al. [39] reported the fatty acid composition of pellets from five US varieties, identifying linoleic acid as the most abundant (30–36%), followed by palmitic acid (16–18%). While linolenic and palmitic acids were the principal PUFA and SFA identified in both studies, the Chilean ecotypes—particularly *Valdivia*—were characterized by notably high levels of eicosenoic acid. This finding is of particular interest, as eicosenoic acid (gondoic acid) has been associated with anti-inflammatory activity by reducing oxidative stress and inhibiting signaling pathways involved in the production of pro-inflammatory mediators [40]. Such properties strengthen the nutraceutical relevance of the lipid profile observed in *Valdivia*, positioning it as a promising candidate for functional food or health-oriented formulations.

Moreover, Li et al. [41] reported that palmitic and linoleic acids also play roles in immune modulation and cellular protection, with palmitic acid linked to macrophage activity and linoleic acid exerting antioxidant and anti-inflammatory effects through the regulation of lipid metabolism and cytokine expression.

The calculated (MUFA + PUFA)/SFA ratios for the *Ranco*, *La Unión*, and *Valdivia* ecotypes were 0.88, 0.85, and 1.00, respectively, reflecting slight variations in the balance between saturated and unsaturated fatty acids among the samples. Although these ratios fall below the Food and Agriculture Organization (FAO) and World Health Organization (WHO) recommended threshold (>1.6) for dietary lipids, and the ω-3/ω-6 ratio of 0 indicates a predominance of ω-6 fatty acids, it should be emphasized that hops are not typically consumed as a primary lipid source. Instead, their nutritional and functional relevance lies in their rich composition of bioactive compounds, including polyphenols, flavonoids, bitter acids, and xanthohumol, which are known for their antioxidant, antimicrobial, and anti-inflammatory properties [2,7,10,11].

According to Zhou et al. [42], the ideal dietary fatty acid profile should feature a substantial amount of MUFA with appropriate ratios associated with enhanced nutritional quality. In this context, while the lipid profile of Chilean hop ecotypes does not confer a direct nutritional advantage compared to conventional dietary lipid sources, Valdivia exhibits a more balanced (MUFA + PUFA)/SFA ratio (1.00), suggesting a higher proportion of unsaturated fatty acids. More importantly, the overall phytochemical profile of these ecotypes supports their value as sources of bioactive molecules with multifunctional potential for the food, pharmaceutical, and cosmetic industries, where health benefits are derived mainly from secondary metabolites rather than from fatty acid composition alone.

### 3.4. Alpha and Beta Acids Content

The composition of α-acids and β-acids in the Chilean hop ecotypes showed statistically significant variability (Table 4), with the *Valdivia* ecotype standing out for its higher levels in all the parameters evaluated. The total α-acids content was highest in *Valdivia* (2.87 ± 0.07% *w*/*w* dry matter), followed by *La Unión* (2.68 ± 0.21% *w*/*w* dry matter) and *Ranco* (2.31 ± 0.03% *w*/*w* dry matter). Among the individual components, cohumulone and adhumulone also reached their highest values in *Valdivia*, with 0.71 ± 0.01% *w*/*w* d.m. and 2.16 ± 0.06% *w*/*w* d.m., respectively.

In terms of β-acids, *Valdivia* again presented the highest total concentration (6.49 ± 0.21% *w*/*w* dry matter), while *La Unión* and *Ranco* showed similar values (5.59 ± 0.17% *w*/*w* d.m. and 5.63 ± 0.12% *w*/*w* d.m., respectively). The specific compounds colupulone and adlupulone also reflected this trend, with *Valdivia* registering values of 2.88 ± 0.08% *w*/*w* d.m. and 3.60 ± 0.12% *w*/*w* d.m., significantly exceeding the other ecotypes. It has been demonstrated that hop bitter acids can be effective against resistant Gram-positive pathogens, such as methicillin-resistant *Staphylococcus aureus* (MRSA), by inhibiting protein and bacterial DNA synthesis. The stability of β-acids during storage also favors their use in antimicrobial formulations, increasing their applicability in the pharmaceutical and food industry [43].

Nesvadba et al. [44] reported a wide range of α-acids (2.96–14.03% *w*/*w*) and β-acids (2.77–7.30% *w*/*w*) contents in European hop varieties, whereas the Chilean ecotypes analyzed in this study exhibited less variability. Although the β-acids of the Chilean ecotypes are comparable to the higher values of the European varieties, the α-acids are significantly lower, suggesting a more balanced profile between both compounds in the Chilean ecotypes that could be considered more like an equivalent to a landrace variety. This could be attributed to both their genetic background and the homogeneous agroclimatic conditions of their growing areas.

Despite the more balanced values of the Chilean ecotypes, these results highlight a higher concentration of bioactive compounds in the *Valdivia* ecotype, suggesting its superior potential for industrial applications related to the bitterness and antimicrobial properties of hop extracts.

### 3.5. Xanthohumol Content

**Figure 2:**
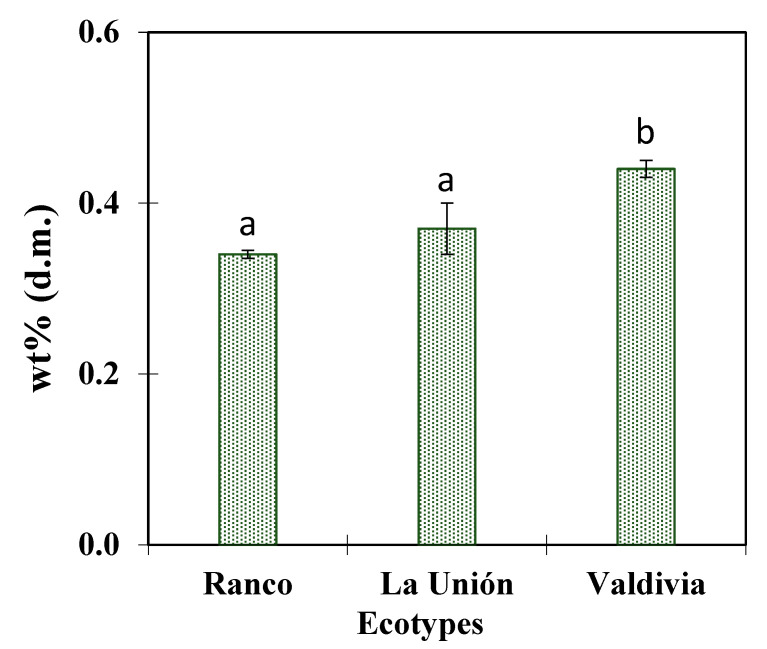
Xanthohumol content in different samples of Chilean hop ecotypes expressed in weight % on a dry matter basis (mean ± standard deviation). On the bars, different letters (a,b) indicate significant differences as per Multiple Range Test (MRT) (*p* < 0.05).

Xanthohumol stands out for its high bioactivity as a rich source of phenolic compounds with recognized functional properties. Considering its nutraceutical potential, the xanthohumol content was evaluated in three Chilean ecotypes (*Ranco*, *La Unión*, and *Valdivia*), to identify intraspecific variability that would allow the selection of germplasms with greater functional value. The results in Figure 2 show the mean xanthohumol concentration expressed as a percentage by weight (Wt%) on a dry matter basis, and its corresponding standard error.

According to the quantified values, the *Valdivia* ecotype presented the highest xanthohumol content (~0.44% d.m.), followed by *La Unión* (~0.37% d.m.) and *Ranco* (~0.34% d.m.). Despite these numerical differences, the statistical analysis revealed no significant differences among the ecotypes, suggesting homogeneity in the metabolite concentration among the evaluated ecotypes. The error bars, notoriously narrow in all cases, reflect low intra-ecotype variability and high reproducibility of the measurements, which reinforces the robustness of the data. The absence of significant differences may be explained by genetic background and a convergence in the factors regulating prenylated compound biosynthesis in these varieties including the shared edaphoclimatic environment [45]. The observed homogeneity could be associated with the genetic and the fact that all ecotypes were grown under similar environmental conditions in southern Chile, which would have attenuated the differential expression of genes involved in xanthohumol biosynthesis.

A study in Central Europe has shown that varieties such as Ruslan and Xanthus can reach concentrations of up to 1% dry weight under optimized growing and postharvest conditions [46]. In comparison, the values obtained in Chilean ecotypes are within the lower range of these international references, although they still represent biologically relevant concentrations for industrial applications. On the other hand, previous studies demonstrated [14] that internationally established varieties such as *Cascade* and *Kazbek* yield less in xanthohumol accumulation when compared to Chilean ecotypes. This finding suggests that native ecotypes grown under edaphoclimatic conditions in southern Chile not only equal but may even surpass the functional potential of commercial cultivars widely used in Europe and North America.

Despite the absence of significant differences, the identification of stable and relatively high levels of xanthohumol in the three ecotypes evaluated is promising, especially considering the growing demand for natural ingredients with functional properties. The quantified values position these materials as valid candidates for breeding programs aimed to develop new varieties with a high bioactive load, as well as for the development of standardized extracts to be used in functional foods, supplements or pharmaceutical formulations. Future research could investigate the influence of phenological stage, agronomic practices, and extraction methods on xanthohumol accumulation to optimize its commercial use.

### 3.6. Antioxidant Capacity

The antioxidant capacity of hop extracts from three Chilean ecotypes (*Ranco*, *La Unión*, and *Valdivia*) was evaluated using the DPPH, FRAP and ABTS assays, and the results are shown in Table 5. In the DPPH assay, the *Valdivia* ecotype presented the highest value of antioxidant capacity with 14.67 ± 0.12 mM Trolox/g d.m., significantly higher compared to the other two ecotypes. *Ranco* (13.05 ± 0.05 mM Trolox/g d.m.) and *La Unión* (12.69 ± 0.02 mM Trolox/g d.m.) had similar values, with no significant differences between them. In the FRAP assay, *Valdivia* also showed superior performance (10.10 ± 0.42 mM Trolox/g d.m.), followed by *Ranco* (9.52 ± 0.18 mM Trolox/g d.m.) and *La Unión* (8.22 ± 0.21 mM Trolox/g d.m.). Similarly, in the ABTS assay, *Valdivia* stood out with 10.00 ± 0.18 mM Trolox/g d.m., while *Ranco* (9.19 ± 0.03 mM Trolox/g d.m.) and *La Unión* (9.09 ± 0.07 mM Trolox/g d.m.) did not show significant differences between them. This variability in antioxidant activity could be related to external factors. Furthermore, it has been shown that differences in antioxidant activity between different hop genotypes can be significant, even when compared under the same environmental conditions [47].

Antioxidant compounds, such as phenols and flavonoids present in hop extracts, have been shown to play a key role in inhibiting pathogenic microorganisms [48]. Their ability to generate oxidative stress in bacteria through the production of reactive oxygen species (ROS) can compromise the integrity of bacterial cell membranes and disrupt essential processes, such as DNA replication and protein synthesis [49].

In this study, it was observed that hop extracts, with their high antioxidant capacity, have dual potential: they not only offer protection against oxidative stress [50], but can also enhance direct antimicrobial activity by destabilizing the antioxidant defenses of resistant bacteria [51,52]. In particular, the *Valdivia* ecotype, with the highest antioxidant activity values, could play a more prominent role in these interactions due to the high concentration of bioactive compounds present in its extracts.

In summary, these findings suggest that the correlation between antioxidant activity and antimicrobial properties of hop extracts highlights their applicability in the fight against infections caused by multidrug-resistant microorganisms.

### 3.7. Antimicrobial Activity

To eliminate the impact of the extract solvent on antimicrobial activity, each extract was dried and then resuspended in sterile distilled water. Table 6 presents the MIC values of each extract evaluated (*Ranco*, *La Unión*, and *Valdivia*) against four multidrug-resistant clinical isolates (*K. pneumoniae*, *E. coli*, *P. aeruginosa*, and *E. faecalis*) as well as their ATCC strains. The results are expressed in milligrams per milliliter (mg/mL). Determining the MIC allowed us to establish the minimum concentration required of hop extract to inhibit bacterial growth and to compare the efficacy of the extracts across different ecotypes.

The results indicate that, regardless of their susceptibility profile, most bacterial strains exhibit a MIC of 5 mg/mL. However, significant differences were observed in the susceptibility of *E. faecalis* ATCC 29212 and vancomycin-resistant *E. faecalis*, particularly in the *Valdivia* ecotype, which demonstrated a significantly lower MIC of 2.5 mg/mL. This finding suggests that the extract from this ecotype may exhibit enhanced inhibitory activity against some Gram-positive bacteria. These findings are consistent with recent reports [51,53,54] indicating that hop extracts tend to exert stronger antimicrobial activity against Gram-positive with respect to Gram-negative bacteria. In a recent study, Piasecki et al. [55], the evaluated the antimicrobial activity of methanolic extracts from various Polish hop varieties against Gram-positive bacteria, reporting that the xanthohumol-rich fraction exhibited strong activity effects, showing a minimum inhibitory concentration (MIC) of 3.9 µg/mL against methicillin-resistant *Staphylococcus aureus* and moderate activity against *Enterococcus faecalis strains*. These MIC differences indicate that methanolic extracts, due to their greater efficiency in extracting xanthohumol and other prenylated flavonoids, exhibit higher antibacterial activity compared to the aqueous extracts used in the present study.

Another relevant observation is that carbapenem-resistant *P. aeruginosa* also shows a MIC of 2.5 mg/mL for the *Valdivia* ecotype, while the other ecotypes require twice the concentration to inhibit bacterial growth. This result suggests that certain compounds in the *Valdivia* extract possess a more pronounced antimicrobial activity against this multidrug-resistant strain. In contrast, *E. coli* ATCC 25922, *K. pneumoniae* ATCC 700603 and their carbapenem-resistant variants showed no differences in MICs between ecotypes, remaining constant at 5 mg/mL. This behavior indicates a lower sensitivity of Gram-negative to hop extracts compared to *E. faecalis* and *P. aeruginosa*, suggesting differences in the mechanisms of action of the antimicrobial compounds present in the extracts.

Research on different hop genotypes has shown that their antimicrobial activity varies significantly depending on the chemical composition [56]. Extracts of different hop varieties were evaluated and found that the concentrations of xanthohumol and α and β acids are directly correlated with antimicrobial efficacy against certain microorganisms. These findings align with the present results, in which aqueous extracts from Chilean hop ecotypes generally displayed uniform antimicrobial activity, with notable exceptions observed for certain clinically relevant strains. The results suggest that the chemical profile of the *Valdivia* ecotype could contain bioactive compounds with higher antimicrobial potential against resistant *E. faecalis* and *P. aeruginosa,* which warrants further evaluation of its chemical composition.

Although the MIC results obtained in the present study are outside the optimal antimicrobial range established for plant extracts (<100 μg/mL) according to the criteria described in [57], the antimicrobial activity observed provides evidence of the antimicrobial potential of these Chilean ecotypes, which could serve as a basis for optimization studies focused on extraction methodology and formulation strategies in order to improve the bioactive potential of these hop ecotypes.

### 3.8. Anti-Tumoral Activity

**Figure 3:**
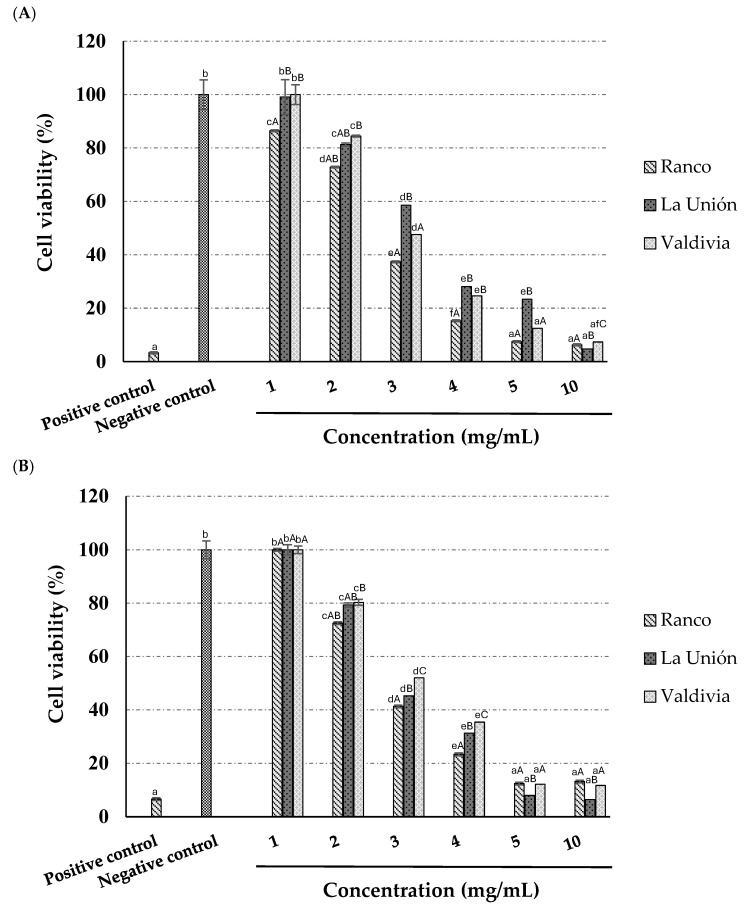
Effect on cell viability of Chilean hop ecotypes on cancer cell lines. Cell viability percentage of H1299 (**A**) and MCF-7 (**B**) cells treated with positive control (50% dimethylsulphoxide; DMSO), negative control (with vehicle) and different concentrations of Chilean hop ecotypes for 48 h, evaluated by MTT assay. Values are reported as mean ± standard deviation (*n* = 3). The same letters, lower case among concentrations of the same ecotype and controls, and upper case between ecotypes at the same concentration, do not significantly differ (*p* < 0.05).

Figure 3 shows the anti-tumoral activity of two cell lines, H1299 (A), corresponding to non-small cell lung carcinoma, and MCF-7 (B), derived from mammary carcinoma, after 48 h of exposure to different concentrations of Chilean hop ecotypes. The evaluation of cell viability by MTT assay allowed us to quantify the impact of treatment with different concentrations of Chilean hop ecotypes on the two cell lines studied.

The evaluation of the effect of Chilean hop ecotypes on cell viability in the H1299 and MCF-7 lines by MTT assay revealed significant concentration- and ecotype-dependent differences. In line H1299 (Figure 3A), cell viability decreased significantly as the concentration of the extract of the different ecotypes increased. Higher concentrations significantly reduced cell viability compared to the negative control, with statistically significant differences between the ecotypes at the same concentration (*p* < 0.05). The 10 mg/mL concentration in the ecotype *La Unión* had the highest ability to reduce cell viability. One of the ecotypes more pronounced cytotoxic activity suggests the presence of bioactive compounds with a more significant antiproliferative effect on this cell type.

In the MCF-7 cell line (Figure 3B), cell viability was reduced following treatment with the hop ecotypes, although the magnitude of the effect was smaller compared to the H1299 line. Exposure to higher concentrations resulted in a significant decrease in cell viability relative to the negative control, but there were less marked differences between the ecotypes. These variations in cellular response suggest differential sensitivity between the cell types tested.

The antiproliferative effect of hop extracts has been studied in several cancer cell lines, such as human hepatoma carcinoma (Hep3B) and human colon carcinoma (HT-29), with antitumoral activity at similar concentrations (around 1 mg/mL) [58].

*Humulus lupulus* has many flavonoids with relevant antitumoral activity, one of them with high potential in the prevention and treatment of cancer is xanthohumol [58], a prenylated chalcone present in hop, which possesses prominent activities against different diseases, including cancer [11,59,60]. Interestingly, the results obtained in this study show stable and relatively high levels of xanthohumol in the three ecotypes analyzed, which could be associated with the inherent antitumor activity.

Indeed, xanthohumol has been the most effective prenylflavonoid (IC_50_ ranging from 3.6 to 7.3 mM), decreasing cell survival in colorectal cancer cell lines SW480, SW620 and CaCo-2, with a relevant impact on the activity of caspases, and ROS formation [61].

## 4. Conclusions

This study provides new insights into the nutritional and bioactive potential of Chilean hop ecotypes (*Ranco*, *La Unión*, and *Valdivia*), highlighting their distinctive chemical composition and functional properties. Compositional characterization revealed clear differences among the three ecotypes: the *Valdivia* ecotype exhibited higher levels of carbohydrates, minerals, functional fatty acids (MUFA/PUFA) and bitter acids; the *La Unión* ecotype presented high protein levels; and the *Ranco* ecotype showed exceptionally high amino acid concentrations that triple those of commercial varieties, while all ecotypes maintained stable levels of xanthohumol.

All ecotypes displayed relevant bioactivities, but with marked differences. The *Valdivia* ecotype stood out for its superior antioxidant capacity, higher concentrations of bitter acids and xanthohumol, and enhanced antimicrobial activity against multidrug-resistant strains such as *E. faecalis* and *P. aeruginosa.* The extracts also induced a dose-dependent reduction in the viability of human cancer cell lines, particularly H1299, suggesting potential antiproliferative effects.

Overall, these findings highlight the functional diversity of Chilean hop ecotypes and their promise as sources of bioactive compounds. Nevertheless, the results were obtained exclusively under in vitro conditions. Furthermore, we did not determine a complete phytochemical profile of the samples, which may also influence the observed bioactivities. Therefore, further research—including studies on phytochemical characterization, bioavailability, metabolism, safety, and extraction scalability—is necessary to validate their nutraceutical relevance and to assess their feasibility for future applications in the food, pharmaceutical, and cosmetic industries.

## Figures and Tables

**Table 1:** Proximal composition of different samples of Chilean hop ecotypes.

Ecotype	Moisture (% d.m.)	Ash (% d.m.)	Fat (% d.m.)	Protein (% d.m.)	Total Carbohydrates (% d.m.)
*Ranco*	5.45 ± 0.01 ^c^	7.39 ± 0.07 ^a^	1.90 ± 0.02 ^a^	13.14 ± 0.11 ^b^	77.58 ± 0.12 ^b^
*La Unión*	4.52 ± 0.04 ^b^	7.89 ± 0.16 ^b^	2.37 ± 0.10 ^b^	14.02 ± 0.26 ^c^	75.71 ± 0.20 ^a^
*Valdivia*	3.63 ± 0.02 ^a^	8.05 ± 0.09 ^c^	1.94 ± 0.04 ^a^	11.12 ± 0.18 ^a^	78.90 ± 0.18 ^c^

d.m: dry matter. Different letters in columns (^a–c^) indicate statistically significant differences. Values differ significantly at *p* < 0.05. Values are reported as mean ± standard deviation (*n* = 3).

**Table 2:** Amino acids of different samples of Chilean hop ecotypes.

Ecotype	*Ranco* (g/100 g d.m.)	*La Unión* (g/100 g d.m.)	*Valdivia* (g/100 g d.m.)
ASP	1.80 ± 0.26 ^a^	1.70 ± 0.06 ^a^	1.35 ± 0.55 ^a^
GLU	0.65 ± 0.01 ^a^	0.52 ± 0.00 ^a^	0.48 ± 0.22 ^a^
SER	0.31 ± 0.63 ^a^	0.29 ± 0.06 ^a^	0.30 ± 0.06 ^a^
HIS	0.06 ± 0.11 ^a^	0.14 ± 0.00 ^a^	0.13 ± 0.00 ^a^
GLY	0.25 ± 0.16 ^a^	0.25 ± 0.05 ^a^	0.32 ± 0.05 ^a^
THR	0.39 ± 0.18 ^a^	0.47 ± 0.59 ^a^	0.60 ± 0.01 ^a^
ARG	0.27 ± 0.01 ^a^	0.27 ± 0.00 ^a^	0.27 ± 0.00 ^a^
ALA	0.25 ± 0.08 ^a^	0.31 ± 0.08 ^a^	0.28 ± 0.04 ^a^
TYR	0.14 ± 0.00 ^a^	0.13 ± 0.00 ^a^	0.13 ± 0.00 ^a^
CY2	1.50 ± 0.23 ^a^	1.45 ± 0.22 ^a^	1.14 ± 0.24 ^a^
MET	0.24 ± 0.06 ^a^	0.15 ± 0.06 ^a^	0.15 ± 0.07 ^a^
PHE	0.32 ± 0.08 ^a^	0.24 ± 0.00 ^a^	0.19 ± 0.08 ^a^
ILE	0.57 ± 0.08 ^a^	0.49 ± 0.08 ^a^	0.50 ± 0.00 ^a^
LEU	0.07 ± 0.03 ^a^	0.10 ± 0.01 ^a^	0.10 ± 0.00 ^a^
LYS	0.77 ± 0.05 ^b^	0.55 ± 0.02 ^ab^	0.48 ± 0.10 ^a^

Key: (ASP) aspartate; (GLU) glutamate; (SER) serine; (HIS) histidine; (GLY) glycine; (THR) threonine; (ARG) arginine; (ALA) alanine; (TYR) tyrosine; (CY2) Cystine; (MET) methionine; (PHE) phenylalanine; (ILE) isoleucine; (LEU) leucine; (LYS) lysine. Different letters in rows (^a,b^) indicate statistically significant differences. Values differ significantly at *p* < 0.05. Values are reported as mean ± standard deviation (*n* = 3).

**Table 3:** Comparison of SFA (saturated fatty acids), MUFA (monounsaturated fatty acids) and PUFA (polyunsaturated fatty acids) present in different samples of Chilean hop ecotypes. Results expressed in % (g/100 g d.m.).

Fatty Acids	Ecotype		
	*Ranco*	*La Unión*	*Valdivia*
Undecanoic	5.26 ± 0.01 ^a^	5.17 ± 1.21^a^	5.00 ± 0.05 ^a^
Palmitic	33.35 ± 0.18 ^a^	33.85 ± 1.48 ^a^	30.67 ± 1.26 ^a^
Stearic	19.88 ± 0.53 ^a^	17.32 ± 2.13 ^a^	16.12 ± 1.70 ^a^
Total SFA	53.22 ± 0.35 ^a^	56.35 ± 2.23 ^a^	51.78 ± 2.91 ^a^
Linoleic	16.13 ± 1.17 ^a^	16.31 ± 0.84 ^a^	17.76 ± 0.47 ^a^
Eicosenoic	31.41 ± 0.83 ^a^	31.86 ± 1.74 ^a^	35.83 ± 2.45 ^a^
Total MUFA and PUFA	46.95 ± 0.83 ^a^	48.17 ± 2.23 ^a^	51.86 ± 2.91 ^a^
(MUFA PUFA)/SFA	0.88	0.85	1.00
ω-3/ω-6	0	0	0

Different letters (^a^) in the same row indicate that the values are significantly different (*p* < 0.05). Values are reported as mean ± standard deviation (*n* = 3).

**Table 4:** Acid and beta composition of different samples of Chilean hop ecotypes.

Ecotype	Cohumulone (%*w*/*w*)	Adhumulone (%*w*/*w*)	Total Alpha Acids (%*w*/*w*)	Colupulone (%*w*/*w*)	Adlupulone (%*w*/*w*)	Total Beta Acids (%*w*/*w*)
*Ranco*	0.56 ± 0.01 ^a^	1.75 ± 0.02 ^a^	2.31 ± 0.03 ^a^	2.49 ± 0.05 ^a^	3.14 ± 0.06 ^a^	5.63 ± 0.12 ^a^
*La Unión*	0.65 ± 0.05 ^b^	2.03 ± 0.16 ^b^	2.68 ± 0.21 ^b^	2.50 ± 0.08 ^a^	3.10 ± 0.09 ^a^	5.59 ± 0.17 ^a^
*Valdivia*	0.71 ± 0.01 ^c^	2.16 ± 0.06 ^b^	2.87 ± 0.07 ^b^	2.88 ± 0.08 ^b^	3.60 ± 0.12 ^b^	6.49 ± 0.21 ^b^

Different letters in columns (^a–c^) indicate statistically significant differences. Values differ significantly at *p* < 0.05. Values are reported as mean ± standard deviation (*n* = 3). All results expressed on a dry matter basis.

**Table 5:** Antioxidant capacity determined by the DPPH, FRAP and ABTS assays of different samples of Chilean hop ecotypes.

Hop Ecotypes	mM Trolox/g d.m.		
	DPPH	FRAP	ABTS
*Ranco*	13.05 ± 0.05 ^a^	9.52 ± 0.18 ^a^	9.19 ± 0.03 ^a^
*La Unión*	12.69 ± 0.02 ^a^	8.22 ± 0.21 ^b^	9.09 ± 0.07 ^a^
*Valdivia*	14.67 ± 0.12 ^b^	10.10 ± 0.42 ^c^	10.00 ± 0.18 ^b^

Different letters in columns (^a–c^) indicate statistically significant differences. Values differ significantly at *p* < 0.05. Values are reported as mean ± standard deviation.

**Table 6:** Minimum inhibitory concentration of extract obtained from Chilean hop ecotypes.

Microorganism	mg/mL		
	*Ranco*	*La Unión*	*Valdivia*
*K. pneumoniae* ATCC 700603	5.0	5.0	5.0
*K. pneumoniae* R-carbapenem	5.0	5.0	5.0
*E. faecalis* ATCC 29212	5.0	5.0	2.5
*E. faecalis* R-vancomicina	2.5	2.5	2.5
*P. aeruginosa* ATCC 27853	5.0	5.0	5.0
*P. aeruginosa* R-carbapenem	5.0	5.0	2.5
*E. coli* ATCC 25922	5.0	5.0	5.0
*E. coli* R-carbapenem	5.0	5.0	5.0

R = resistance; ATCC = American Type Culture Collection.

## Data Availability

The original contributions presented in this study are included in the article. Further inquiries can be directed to the corresponding author.
